# Treatment of Indolent Ulcers

**Published:** 1904-06

**Authors:** 


### Treatment of Indolent Ulcers.

STEWART, H. KNOX, Philadelphia, Pa., says: A lady about 40 years of age, suffered from a condition in which both legs, from knees to ankles, were encrusted with a thick scale or scab, which on the surface resembled both in color and appearance the ashy gray hue of the surface of a hornet's nest. I had little hope of relieving her; however, I thought that as glyco-thymoline had given me successful results in previous cases of leg ulcers and obstinate skin conditions, I would try it and accordingly began by spraying the affected parts thoroughly with equal parts of glyco-thymoline and water. These applications were made daily and the legs wrapped also in gauze saturated with 50 per cent. solution of the remedy. The crust began to soften with this line of treatment and was removed little by little. Finally, the entire surface became clean. The appearance then was as if raw flesh had been coated with collodion. The legs were loosely bandaged with gauze kept saturated with 50 per cent. solution of glyco-thymoline. Improvement now became rapid and ultimately a complete cure was obtained, the skin becoming healthy and smooth. The duration of treatment was about nine months. There is no doubt in my mind that glyco-thymoline is distinctly alterative, that it increases cell activity, and is especially useful in indolent conditions, like those described.

